# The utilization of humanized mouse models for the study of inhibitors in HTLV-1 infection

**DOI:** 10.1186/1742-4690-8-S1-A28

**Published:** 2011-06-06

**Authors:** Rachel Van Duyne, Irene Guendel, Kylene Kehn-Hall, Mohammed Saifuddin, Fatah Kashanchi

**Affiliations:** 1 George Mason University, Department of Molecular and Microbiology, National Center for Biodefense and Infectious Diseases, Manassas, VA, 20110, USA; 2 The George Washington University Medical Center, Department of Microbiology, Immunology, and Tropical Medicine, Washington, DC, 20037, USA; 3 CONRAD, Eastern Virginia Medical School, Arlington, VA, 22209, USA

## 

The development of novel techniques and systems to study human infectious diseases in both an in vitro and in vivo settings is always in high demand. Ideally, small animal models are the most efficient method of studying human afflictions. This is especially evident in the study of the human retroviruses, HIV-1 and HTLV-1, in that current simian animal models, though robust, are often expensive and difficult to maintain. Recently significant advances have been made to use human stem cells in immunocomprimised animals and follow the course of infection. HTLV-1-infected humanized non-obese diabetic severe combined immunodeficiency (HU-NOD/SCID) mice have been shown by inoculation of NOD/SCID mice with CD34(+) hematopoietic progenitor and stem cells (CD34(+) HP/HSCs) infected ex vivo with HTLV-1 [1]. These mice exclusively develop CD4 (+) T-cell lymphomas with characteristics similar to ATL and elevated proliferation of infected human stem cells in the bone marrow were observed in mice developing malignancies. We will discuss the results of a panel of inhibitors against NFkB, cyclin/cdk complexes and Jack/Stat pathway that effectively inhibit HTLV-1 and Tax expression in vivo.
